# Supplementary material for: Mapping Optical Chirality with Single Fluorescent Molecules
Source: Nano Lett. 2026 Jan 20;26(9):3012–7. doi: 10.1021/acs.nanolett.5c05316 (PMC12983358; doi:10.1021/acs.nanolett.5c05316)
Supplement: Supplementary file 1 [file nl5c05316_si_001.pdf]

Mapping Optical Chirality with Single Fluorescent Molecules

Daniel Marx,† Ivan Gligonov,† David Malsbenden,‡ Dominik Wöll,‡ Oleksii Nevskyi,*,† and Jörg Enderlein*,†,¶

†*Third Institute of Physics (Biophysics), Georg August University, 37077 Göttingen, Germany.*

‡*Institute of Physical Chemistry, RWTH Aachen University, 52074 Aachen, Germany.*

¶*Cluster of Excellence "Multiscale Bioimaging: from Molecular Machines to Networks of Excitable Cells" (MBExC), Universitätsmedizin Göttingen, Robert-Koch-Str. 40, 37077 Göttingen, Germany.*

E-mail: oleksii.nevskyi@phys.uni-goettingen.de; jenderl@gwdg.de

Materials and methods

Set-up

Confocal scanning microscopy measurements were performed on a custom-built optical setup. For 640 nm excitation, an 80 MHz white-light laser source (Fianium WhiteLase SC450, NKT Photonics) was employed. The laser beam was coupled into a single-mode fiber (PMC-460Si-3.0-NA012-3APC-150-P, Schäfter + Kirchhoff), which was attached to a fiber coupler (60SMS-1-4-RGBV-11-47, Schäfter + Kirchhoff). After the fiber, the output beam was recollimated by an objective (UPlanSApo 10×/0.40,NA, Olympus). The collimated beam then passed through a clean-up filter (MaxDiode 640/8, Semrock) to suppress undesired

spectral components. To control the polarization, the beam passed through a linear polarizer (LPVISC050, Thorlabs), a half-wave ($\lambda/2$) plate (AHWP05ME-550, Thorlabs), and a quarter-wave ($\lambda/4$) plate (AQWP05ME-550, Thorlabs). The resulting polarization state was verified using a polarimeter (PAX1000VIS/M, Thorlabs). During this measurement, the objective was removed and the polarimeter placed at the position of the objective. For left-handed circular polarization an ellipticity of $(-44.93 \pm 0.04)^\circ$ was measured and for right-handed circular polarization an ellipticity of $(44.93 \pm 0.04)^\circ$. The measured ellipticity averaged over different linear polarizations was $(0.01 \pm 0.04)^\circ$.

A quad-band dichroic mirror (ZT405/488/561/640rpc, Chroma) was used to direct the excitation light into the specimen and separate it from the emission light. The excitation beam passed through a fast laser scanning system (FLIMbee, PicoQuant), which was used to deflect the beam while preserving its focus position at the back focal plane of the objective (UApo N 100×/1.49,NA oil, Olympus). The region of interest and the focal plane were controlled using a manual XY stage (Olympus) and a Z-piezo stage (Nano-ZL100, Mad City Labs), respectively. Fluorescence emission was collected by the same objective and de-scanned by the scanning system. The emission light was focused using a 180mm achromatic lens (TTL180-A, Thorlabs), but for this application the typical pinhole was removed. Later, a long-pass filter (635 LP Edge Basic, Semrock) was placed in the detection path to block residual excitation light. Furthemore, a band-pass filter (BrightLine HC 708/75, Semrock) was further used to reject scattered excitation light. Finally, the emission light was focused onto a single-photon detector (τ-SPAD, PicoQuant) using a 30 mm achromatic lens (AC254-030-A-ML, Thorlabs). The output signal from the detector was recorded using a time-correlated single-photon counting (TCSPC) system (HydraHarp 400, PicoQuant), synchronized with the laser trigger signal. Data acquisition was performed using SymPhoTime 64 software (PicoQuant), which controlled both the TCSPC and scanner systems. Typically, scans were acquired with a virtual pixel size of 50 nm, a dwell time of 2.5 ms/pixel, and a TCSPC time resolution of 16 ps.

Sample preparation

The TDI molecules were embedded in a thin PS film. TDI was chosen for its exceptional brightness and photostability in apolar environments. We gratefully thank BASF SE, and especially Dr. Peter Erk and Dr. Martin Könemann, for the donation of a small amount of the TDI.

Samples were prepared on glass slides (High Precision No.1.5H #0107222 cover glasses, Marienfeld GmbH) that were cleaned for 10 minutes in an ultrasonic bath with a solution containing potassium hydroxide (0.63 mol/L) dissolved in distilled water (18%) and ethanol (82%).This is followed by washing with distilled water and treatment with oxygen plasma (Plasma cleaner, Harrick Plasma). Polymer films were deposited onto the cleaned glass slides by spin-coating a drop of polymer solution (PS, M_w = 45.4 kg/mol, $T_g > 90$ °C, Polymer Source. Inc.) in toluene (0.5% w/w) containing the dye at 0.1 nM concentration at 4000 rpm. The film was then dried over night under vacuum at a temperature of 80 °C.

Pattern matching

To determine unknown parameters P, grid-wise pattern matching is used. Let $\{I_1, I_2..., I_n\}$ represent the stack of experimental images for one molecule and $\{T(P, z_1), T(P, z_2), ..., T(P, z_n)\}$ denote the corresponding stack of theoretical images based on the input parameters P. Here, z_k are known and fixed for all corresponding images I_k. The stacks of theoretical images are calculated for every point in the grid of unknown parameters. Now choose the set of parameters P^* that yields the highest total correlation between the set of theoretical and experimental images:

$$P^* = \arg\max_P \frac{1}{n} \sum_{k=1}^{n} \max_{x,y} \left[\gamma(I_k, T(P, z_k))\right] \tag{1}$$

"γ" refers to the normalized cross-correlation generalized to two-dimensions, which is explicitly defined in equation 2 by Lewis.[1] Assuming that the experimental image is larger than the theoretical image, one obtains a matrix of correlation values, which is used to find

the x-y-position of the molecule, by picking the pixel with the highest correlation. This automatically accounts for small drifts during the measurements.

Parameters for calculations of theoretical patterns

To calculate the theoretical patterns, many parameters are needed as input. The setup specific parameters used for our confocal setup and the given sample are summarized in Table S1. For a schematic of the sample, see the Main Figure 2. For the refractive index n of PS a literature value was used.[2] The film thickness d was determined by atomic force microscopy, see Section "Atomic Force Microscopy and Image Analysis". To reduce the number of fit parameters, it is assumed that the position of the single molecules inside the polymer film is in the center of the polymer film ($z_0 = 15$ nm). Because of the thin film, different positions of the molecule only lead to minor changes in the shape of the calculated pattern. To calculate the pattern shown in Main, only primary spherical aberrations were considered. For the estimation of the amplitude of the primary spherical aberrations A_{Aber}, see Section "Primary spherical aberrations". For a detailed comparison with the ideal case with no aberrations and an approach including also the higher-order secondary spherical aberrations, see Section "Secondary spherical aberrations".

Table S1: Parameters used in the theoretical calculations to characterize the setup and the sample.

Parameter	value
numerical aperture NA	1.49
pixel size	50 nm
amplitude of primary spherical aberrations A_{Aber}	0.35
amplitude of secondary spherical aberrations B_{Aber}	0
excitation wavelength λ	640 nm
thickness of the polymer film d	30 nm
position of the molecule inside the polymer film z_0	15 nm
refractive index glass n_{glass}	1.5210
refractive index PS n_{PS}	1.588
refractive index air n_{air}	1.0003

The estimated orientations of the molecules #1, #2 and #3 (see Main Figure 3) and the molecules #4, #5 and #6 (see Main Figure 4) are summarized in Table S2. They are the result of the grid-wise pattern matching with the parameters used as input for the depicted theoretical pattern.

Table S2: Determined orientations of the molecules #1, #2, ... #6 and the polarizations they were measured with.

molecule / parameter	#1	#2	#3	#4	#5	#6
in-plane angle α	227°	331°	197°	72°	196°	252°
out-of-plane angle β	61°	75°	85°	15°	45°	85°
measured polarizations	L-circ.	R-circ.	$\Psi = 82°$	L-circ<br>R-circ<br>$\Psi = 72°$<br>$\Psi = 162°$	L-circ<br>R-circ<br>$\Psi = 16°$<br>$\Psi = 106°$	L-circ<br>R-circ<br>$\Psi = 72°$<br>$\Psi = 162°$

Influence of the individual parameters

All the different parameters impact the shape of the pattern. To give an idea of the influence of the parameters on the the pattern, Figure S1 **A** shows, for the example of molecule #4, the theoretical pattern calculated for a set of parameter variations. One can see that the film thickness has a greater influence on the pattern, when the molecules are positioned closer to the substrate. The theoretical framework can also predict the pattern for all degrees of ellipticity, not only circular and linear polarization. Figure S1 **B** shows one example, how the pattern change depending on the ellipticity.

Atomic Force Microscopy and Image Analysis

The polymer film thickness was estimated by atomic force microscopy (AFM). Therefore, a small cross was scratched down to the coverglass surface in the middle of the polymer film. All AFM measurements were performed in tapping mode under application of a NanoWizard 3 setup (JPK Instruments, Germany). For imaging, OTESPA tips with a resonance frequency

of 300 kHz and a nominal force constant of 26 N/m were applied (NanoAndMore GmbH, Germany). Images with a resolution of 256 × 256 pixels were recorded along the scratch in the polymer film.

The AFM data was analyzed with the JPKSPM Data Processing software (JPK Instruments, Germany) under application of line leveling and a surface plane fit of first order. The results can be seen in Figure S2. For our sample a height of approximately $d = 30$ nm was found.

Aberrations

The only aberrations accounted for were spherical aberrations, since for our setup they are the most likely ones to occur.

Primary spherical aberrations

In the manuscript, only primary spherical aberrations were considered. They change the phase by their corresponding Zernike polynomial (described in[3,4]):

$$\Phi_{\text{Pri.Sph.}} = A_{\text{Aber}} \cdot \sqrt{5}(6r^4 - 6r^2 + 1) \tag{2}$$

To estimate the primary spherical aberrations in our setup 269 single molecules were measured systematically for a stack of focus positions $z = \{-1$ µm, -0.9 µm, -0.8 µm, …, 1 µm$\}$. For every molecule's z-stack, a grid-wise pattern matching was performed. The following grid was used: for the in-plane angle $\alpha = \{0°, 3°, 6°, \ldots, 360°\}$ and for the out-of-plane angle $\beta = \{0°, 3°, 6°, \ldots, 90°\}$. Additionally, small variations in the focus position z are allowed, shifting the experimental and theoretical images relative to each other: $z_{exp} = z_{theo} + 50\text{ nm} \cdot i_z$, with $i_z \in \{-5, -3, \ldots, 5\}$. Most importantly, the amplitude for the spherical aberrations was varied as well: $A_{\text{Aber}} = \{0, 0.05, \ldots, 1\}$. The result can be seen in Figure S3. It shows a histogram of A_{Aber}, where for each molecule the amplitude was picked, which

resulted in the highest correlation. To avoid that mismatches influence the result (caused for example by overlying molecules), a threshold for the correlation of $C_{threshold} \geq 0.65$ was set. This reduced the number of considered molecules to $N_{mol} = 204$. A Gaussian curve was fitted to the histogram to determine its maximum. This resulted in $A_{\mathrm{Aber}} \approx 0.35$. Since the primary spherical aberrations of the setup should be the same for all measurements, this value was used for all calculated theoretical patterns in the manuscript.

Secondary spherical aberrations

It was also tested whether the theoretical model can be further improved by considering not only primary spherical aberrations but also secondary spherical aberrations. These are described by the following higher-order Zernike polynomial:[4]

$$\Phi_{\mathrm{2nd.Sph.}} = B_{\mathrm{Aber}} \cdot \sqrt{7}(20r^6 - 30r^4 + 12r^2 - 1) \tag{3}$$

To estimate the pair of amplitudes $\mathrm{A_{Aber}}$ and $\mathrm{B_{Aber}}$ for the combination of primary and secondary spherical aberrations, grid-wise pattern matching was performed. For each of the molecules #1, #2 and #3, all combinations of $\mathrm{A_{Aber}} \in \{0, 0.05, \ldots, 1\}$ and $\mathrm{B_{Aber}} \in \{0, 0.05, \ldots, 1\}$ were used as input parameters for the grid-wise pattern matching. Figure S4 depicts the highest found correlation for each of the combination and the mean for the three molecules. The highest average correlation was found for $\mathrm{A_{Aber}} = 0.6$ and $\mathrm{B_{Aber}} = 0.25$. Figure S5 shows the resulting pattern when using these aberrations compared to the case where no aberrations are used and the case where only primary spherical aberrations are considered, as in Main Figure 3. To quantify the match between experiment and theory, Figure S6 shows the calculated 2D-correlations between the three different theoretical approaches and the experimental images for all three molecules and polarizations. It clearly shows, that when no aberrations are considered, the correlations decrease significantly as the objective moves closer to the sample. The model that includes both primary and secondary aberrations performs on average slightly better than the model that only considers primary

spherical aberrations, although the improvement is minor, and for some focal positions, the opposite is true. Overall, one can conclude that including higher-order aberrations can further improve the model, but it is unclear whether this significantly describes the experimental pattern better, while it becomes increasingly difficult to decide on the amplitudes.

Image Acquisition

The general experimental settings used to acquire the different experimental images are summarized in Table S3.

Table S3: Setting and statistics for image acquisition while measuring the molecules #1, #2, ... #6. N_{tot} represents the total number of photons collected from this molecule. Note that some molecules did not bleach during acquisition. Photons from all acquired images are included, not only those shown in any of the figures. Also listed are the number of frames summed per image, the total acquisition time t_{image}, the dwell time, and the distance dz between two consecutive focal positions.

molecule / parameter	#1	#2	#3	#4	#5	#6
N_{tot} [$1e^6$ photons]	≈ 5	≈ 23	≈ 4	≈ 9	≈ 6	≈ 3
Frames per image	100	1000	400	1000	1000	1000
Frame rate [1/s]	25.1	25.7	25.7	28.7	24.4	28.7
Total acquisition time/image t_{image} [s]	4.0	38.9	15.6	34.8	41.0	34.8
Dwell time [μs]	4	3	3	3.5	3.5	3.5
dz [μm]	0.05	0.1	0.1	/	/	/

The images were measured with frame rates of more than 25 frames per seconds. Up to 1000 frames are binned together for the final image. This process ensures that, for example, in the case of short blinking of the dye, the final image is averaged out, leading to a smooth pattern of consistently high quality. The effect of frame binning can be seen in more detail in Figure S7, which shows how the pattern of molecule #2, measured with right-handed circular polarization, appears after binning an increasing number of frames together.

Interestingly, one can see individual events of fast blinking in the case of one frame. At the focus position $z = -0.4$ μm, a row of dark pixels is clearly visible, due to the molecule being in a dark state during these scanning steps. Sometimes, molecules remain in a dark state for

extended periods. This can be observed for the focal position $z = -0.2$ μm. The molecule entered a dark state for multiple frames, resulting in decreased intensity when summing over 1000 frames compared to the other focal positions. Table S3 also contains the total number of collected photons from every molecule, showcasing the very high photostability of the TDI dye, with often more than 5 million photons emitted by a single molecule.

The photon statistics for every image shown in Main Figure 3, and Main Figure 4, are summarized in Table S4 and Table S5, respectively.

Testing the theoretical framework for a different dye: a PDI-G0 derivative

Our framework should be able to predict the pattern for any single-emitter with an absorption dipole. Here, this is demonstrated with a derivative of PDI-G0 (see Figure S8). It was excited with a wavelength of 563 nm, and a band-pass filter (BrightLine HC 617/73, AHF analysentechnik) was used to reject scattered excitation light. Except for these changes, the sample preparation and parameters remained the same. Figure S8 shows the experimental pattern of three PDI molecules measured with left-handed and right-handed circular polarization, as well as two different linear polarizations. Additionally, the molecules were measured with radially and azimuthally polarized laser beams. To generate these, the quarter-wave plate was exchanged with a customized zero-order vortex half-wave retarder (WPV10L-560-SP, Thorlabs). The combination of the two polarizations is widely used for determining single-emitter absorption dipole orientations.[5–8] Azimuthal polarization is especially well-suited for determining the in-plane angle α, while radial polarization can be used to find the out-of plane angle β. The resulting angles are listed in Table S6. These angles were then used to predict the theoretical pattern for the other polarizations, also shown in Figure S8.

Table S4: Statistics for the experimental images shown in Main Figure 3. Listed are the maximum number of photons N_{max}, the average background flux, the background per image, and the average number of photons per image. The background was averaged over a region of pixels with no molecules in the near vicinity. Please note that the total acquisition times t_{image} and the number of summed frames per image differ from each other and are listed in table S3.

molecule	z [μm]	N_{max} [phot.]	background [phot./(px · s)]	background [phot./(px · t_{image})]	N_{avg} [phot./(px · t_{image})]
#1	-1	371	6.37	25	73
#1	-0.8	535	6.56	26	78
#1	-0.6	855	5.98	24	80
#1	-0.4	1129	5.91	24	82
#1	-0.2	1299	5.84	23	82
#1	0	1252	5.80	23	87
#1	0.2	971	5.33	21	97
#1	0.4	544	5.61	22	101
#1	0.6	390	6.12	24	98
#2	-1	2250	3.16	123	439
#2	-0.8	3523	3.21	125	494
#2	-0.6	4727	3.25	126	518
#2	-0.4	5968	3.23	126	550
#2	-0.2	2637	3.28	128	279
#2	0	8121	3.33	130	635
#2	0.2	7272	3.37	131	702
#2	0.4	4218	3.42	133	751
#2	0.6	3055	3.36	131	702
#3	-1	655	3.27	51	140
#3	-0.8	722	3.29	51	153
#3	-0.6	943	3.38	53	166
#3	-0.4	1242	3.15	49	174
#3	-0.2	1458	3.24	51	168
#3	0	1617	3.34	52	174
#3	0.2	1594	3.33	52	185
#3	0.4	1269	3.20	50	195
#3	0.6	784	3.29	51	198

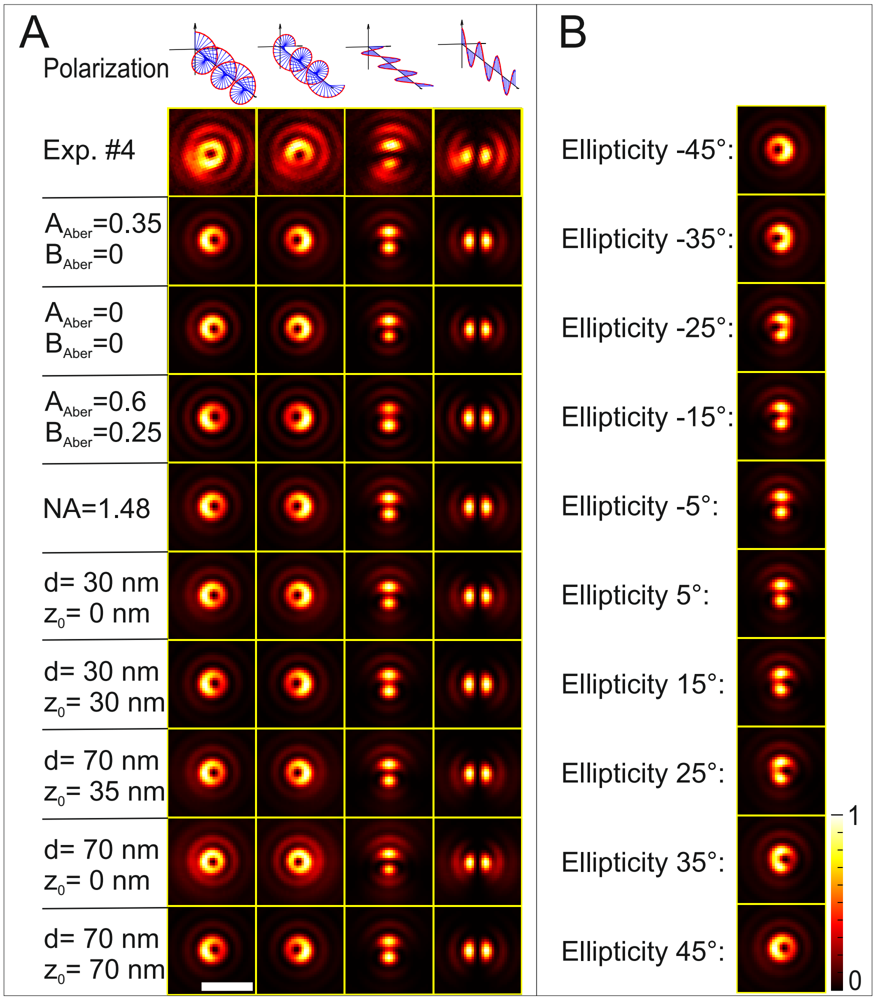

Figure S1: (**A**) Theoretical pattern calculated for a set of different variations of the input parameter for left-handed and right-handed circular polarization, as well as linear polarization parallel and perpendicular to the in-plane angle of the molecule. To compared this with the measurements of molecule #4, the out-of-plane angle was set to $\beta = 15°$, the in-plane angle to $\alpha = 0°$ and the focal position to $z = -0.35$. In each row, only the mentioned parameter is varied from the values listed in Table S1. (**B**): Theoretical pattern for different degrees of ellipticity, from right-handed circular over linear to left-handed circular polarization. For all images, $\Psi = 0°$. All images are normalized by their highest intensity. The scale bar is 1 µm.

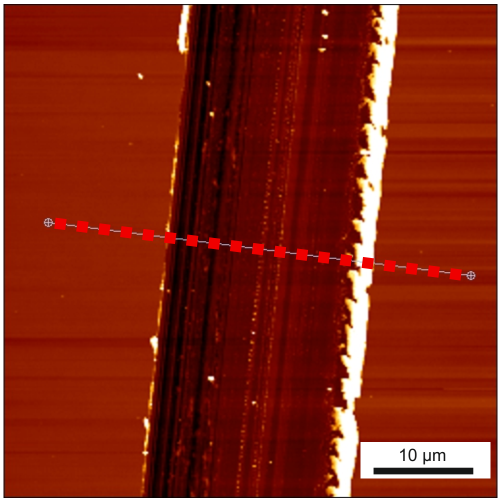

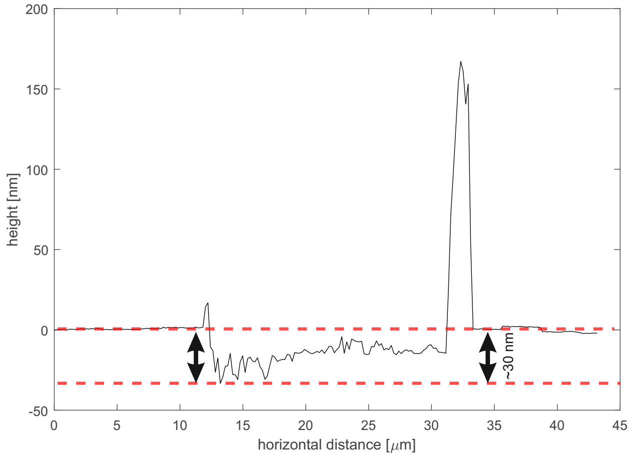

Figure S2: Left: Topography image of a scratch through a polystyrene polymer film, prepared following the described protocol. Right: Height profile along the marked red line. Estimations of the approximate film thickness ($d \approx 30$ nm) by line construction is depicted.

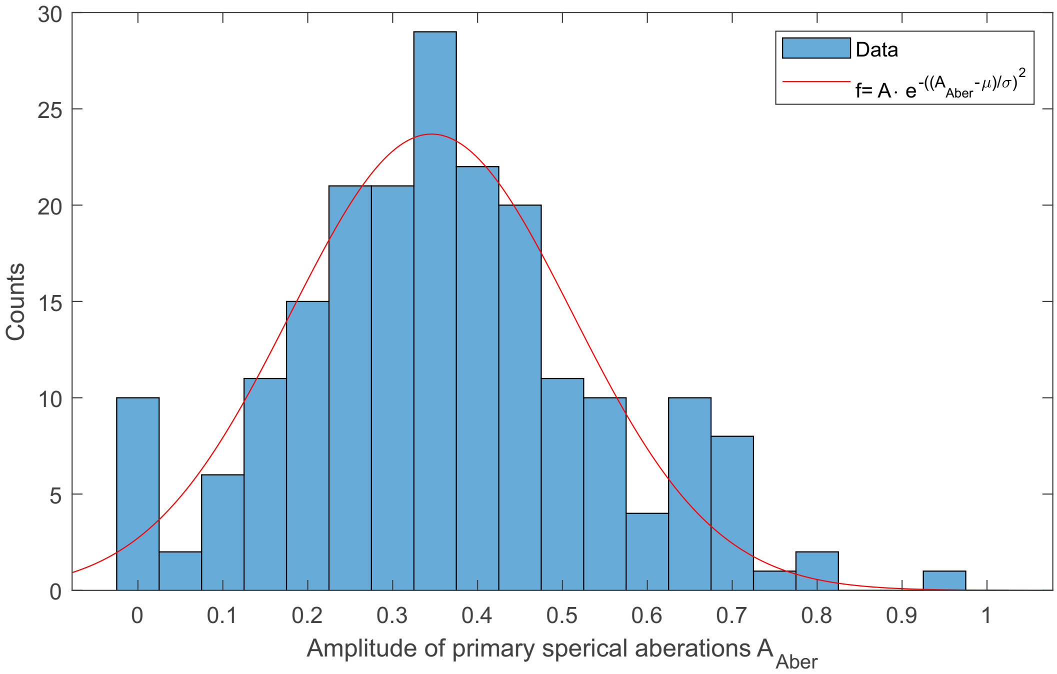

Figure S3: Histogram of the amplitudes of spherical aberrations, which resulted in the best correlation for each of the 204 considered molecules, respectively. A Gaussian fit f was performed to determine the maximum of the distribution. The fit results are $A \approx 24$, $\mu \approx 0.35$ and $\sigma = 0.24$.

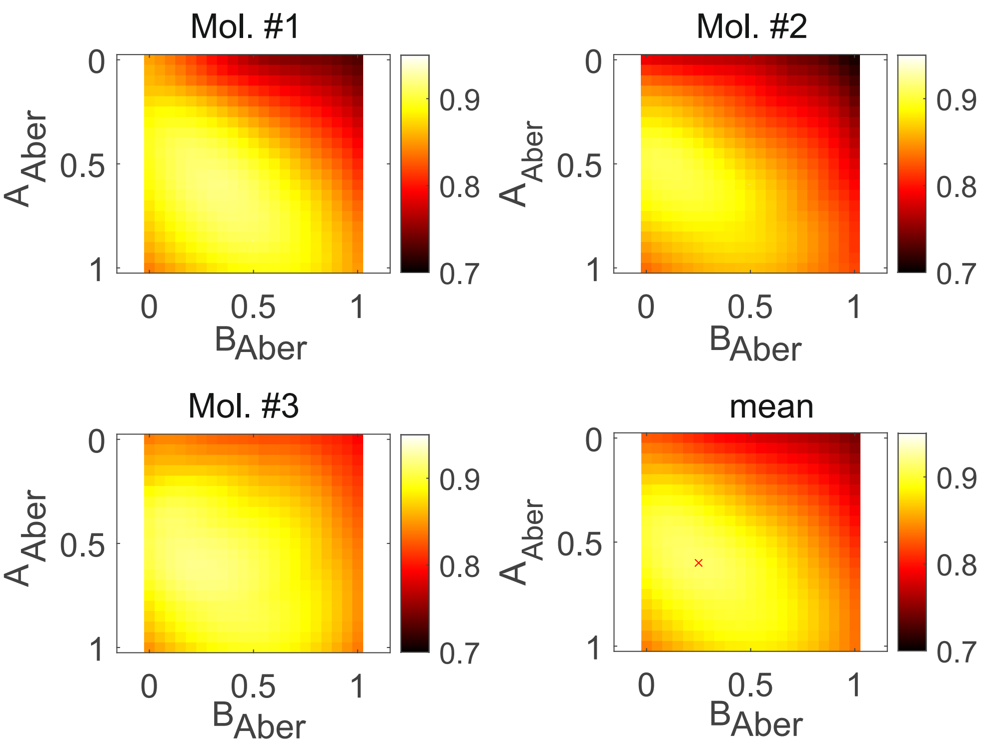

Figure S4: The highest correlation estimated by pattern matching for a grid of varying input parameters A_{Aber} and B_{Aber} compared to the z-stack of the molecules #1, #2 and #3. The bottom-right image shows the mean of the other three images. The red cross marks the position of the overall highest correlation, found at $A_{Aber} = 0.6$ and $B_{Aber} = 0.25$.

Table S5: Statistics for the experimental images shown in Main Figure 4. Listed are the maximum number of photons N_{max}, the average background flux, the background per image, and the average number of photons per image.

molecule	polarization	N_{max} [phot.]	background [phot./(px · s)]	background [phot./(px · t_{image})]	N_{avg} [phot./(px · t_{image})]
#4	L-circ.	1443	3.77	131	205
#4	R-circ	1640	3.70	129	202
#4	$\Psi = \alpha$	2365	3.74	130	202
#4	$\Psi = \alpha + 90°$	2266	3.67	127	202
#5	L-circ.	3622	3.03	124	212
#5	R-circ	3718	3.05	125	202
#5	$\Psi = \alpha$	6137	2.95	121	231
#5	$\Psi = \alpha + 90°$	1129	2.80	115	145
#6	L-circ.	2597	3.85	134	188
#6	R-circ	3111	3.95	137	189
#6	$\Psi = \alpha$	4720	4.02	140	222
#6	$\Psi = \alpha + 90°$	739	3.59	125	137

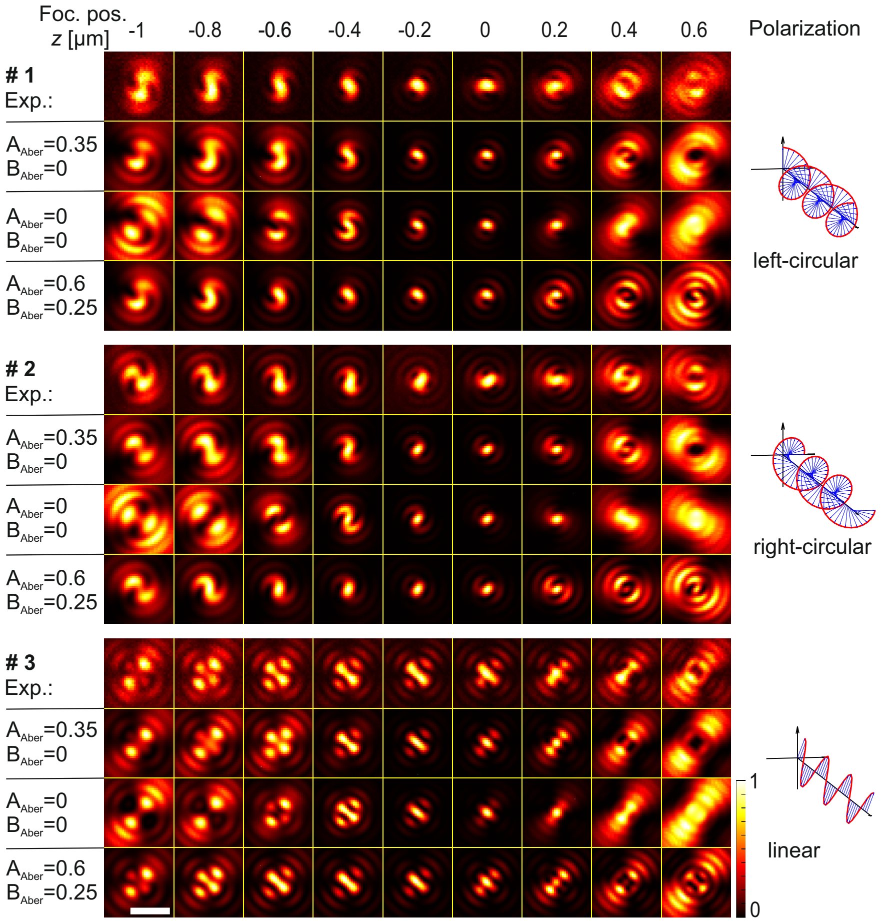

Figure S5: Experimental and corresponding theoretical patterns for three TDI molecules with fixed orientations embedded in a thin PS film. In addition to the theoretical images shown in Main Figure 3, the simulated theoretical patterns for no aberrations and the best match when considering the next higher order of spherical aberrations are illustrated. For each molecule, images were recorded at different focal positions z of the objective. Negative z-values correspond to moving the objective closer to the sample. The first molecule was measured using left-handed circular polarization, the second with right-handed circular polarization, and the third with linear polarization oriented at $\Psi = 82°$. All images are normalized by their respective maximum intensity. For the experimental images, the maximum number of photons are listed in Table S4. The scale bar is 1 µm.

Table S6: Radial and azimuthal polarization were used to determine the orientations of PDI molecules #7, #8, #9. The in-plane angle α was determined using azimuthal polarization and the out-of-plane angle β was determined using radial polarization.

parameter \ molecule	#7	#8	#9
in-plane angle α	90°	193°	5°
out-of-plane angle β	56°	59°	85°

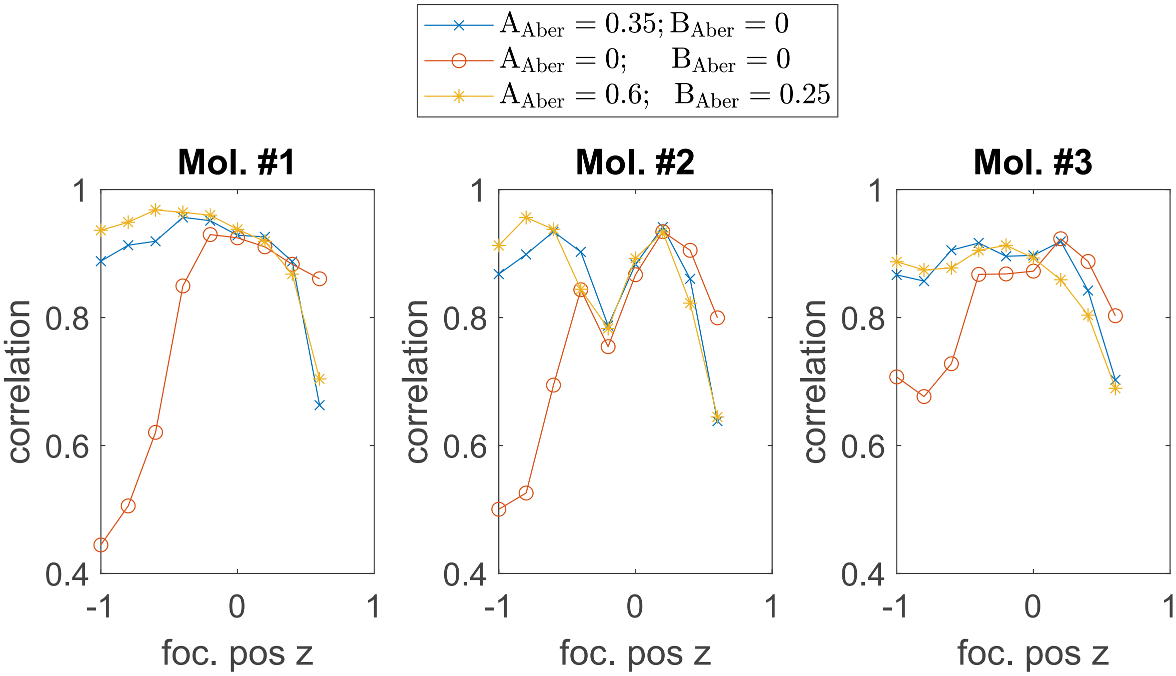

Figure S6: Correlations between the experimental and the three different theoretical pattern calculated for the images taken at the different focal positions. The difference between the theoretical pattern lies in the amplitudes of the two considered aberrations, which were used as the input for the pattern matching. The three pairs of aberrations are [$A_{Aber} = 0.35$, $B_{Aber} = 0$], [$A_{Aber} = 0$, $B_{Aber} = 0$], and [$A_{Aber} = 0.6$, $B_{Aber} = 0.25$]. This is plotted for the three molecules #1, #2, and #3.

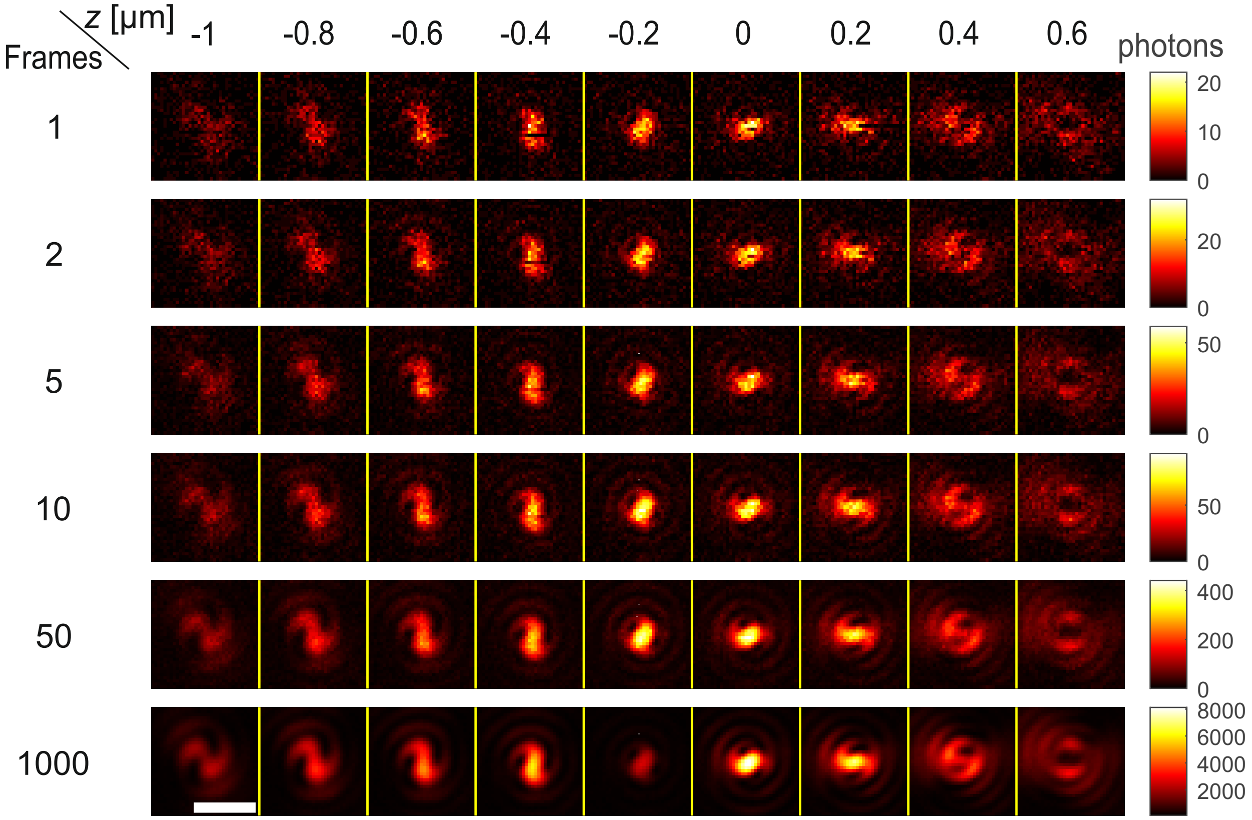

Figure S7: Experimental images of molecule #2, measured with right-handed circular polarization. The images taken at different focal positions z are displayed after binning an increasing number of frames together. Please note that, to provide a better understanding of the number of photons, the images are not normalized, so the color directly represents the photon counts.

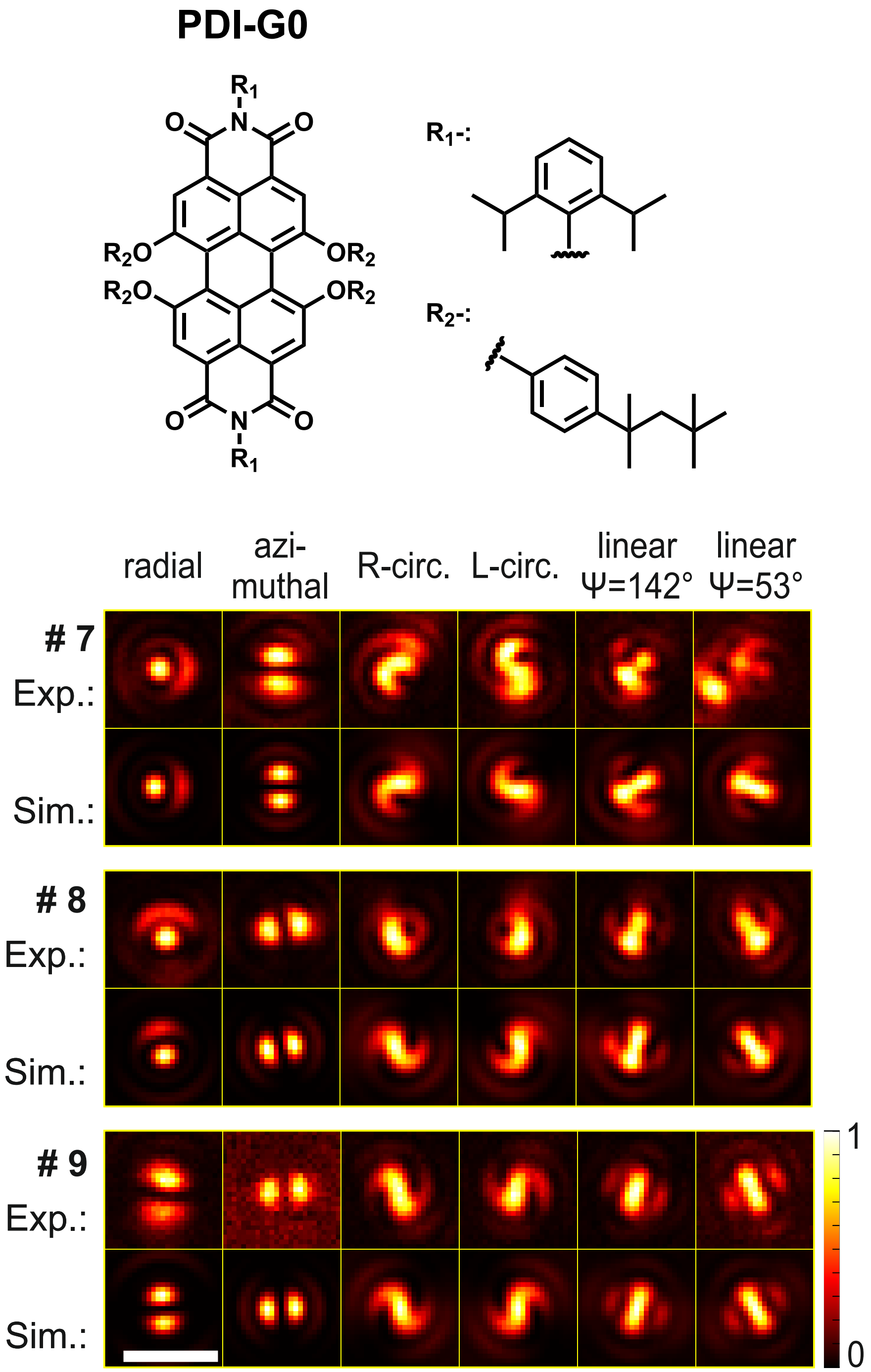

Figure S8: Structure of PDI-G0 molecule and experimental and corresponding theoretical pattern for three PDI-G0 molecules #7, #8, and #9. Their orientations, shown in Table S6, were determined using azimuthal and radial polarization, measured in focus ($z = 0$ µm). These orientations were then used to predict the pattern for right-handed and left-handed circular polarization, as well as for two linear polarizations, measured at $z = -0.35$ µm. The other parameters used for fitting are listed in Table S1, with the only change being the excitation wavelength, which was set to $\lambda = 563$ nm. All images are normalized by their respective maximum intensity. The scale bar is 1 µm.

The data that support the findings of this Letter including routines for pattern calculation and matching are openly available:
https://gitlab.gwdg.de/d.marx/mapping-complex-optical-light-field-distribution-with-single-fluorescence-molecules

References

(1) Lewis, J. P. Fast normalized cross-correlation. Vision interface. 1995; pp 120–123.

(2) James, J.; Unni, A. B.; Taleb, K.; Chapel, J.-P.; Kalarikkal, N.; Varghese, S.; Vignaud, G.; Grohens, Y. Surface engineering of polystyrene–cerium oxide nanocomposite thin films for refractive index enhancement. *Nano-Struct. Nano-Objects* **2019**, *17*, 34–42.

(3) Fazel, M.; Grussmayer, K. S.; Ferdman, B.; Radenovic, A.; Shechtman, Y.; Enderlein, J.; Pressé, S. Fluorescence microscopy: A statistics-optics perspective. *Rev. Mod. Phys.* **2024**, *96*, 025003.

(4) Niu, K.; Tian, C. Zernike polynomials and their applications. *J. Opt.* **2022**, *24*, 123001.

(5) Chizhik, A. I.; Chizhik, A. M.; Khoptyar, D.; Bär, S.; Meixner, A. J. Excitation isotropy of single CdSe/ZnS nanocrystals. *Nano Lett.* **2011**, *11*, 1131–1135.

(6) Karedla, N.; Stein, S. C.; Hähnel, D.; Gregor, I.; Chizhik, A.; Enderlein, J. Simultaneous measurement of the three-dimensional orientation of excitation and emission dipoles. *Phys. Rev. Lett.* **2015**, *115*, 173002.

(7) Ghosh, S.; Chizhik, A. M.; Yang, G.; Karedla, N.; Gregor, I.; Oron, D.; Weiss, S.; Enderlein, J.; Chizhik, A. I. Excitation and emission transition dipoles of type-II semiconductor nanorods. *Nano Lett.* **2019**, *19*, 1695–1700.

(8) Zhang, O.; Zhou, W.; Lu, J.; Wu, T.; Lew, M. D. Resolving the three-dimensional

rotational and translational dynamics of single molecules using radially and azimuthally polarized fluorescence. *Nano Lett.* **2022**, *22*, 1024–1031.
